# A cross-sectional survey of practices and knowledge among antibiotic retailers in Nairobi, Kenya

**DOI:** 10.7189/jogh.09.020412

**Published:** 2019-12

**Authors:** Dishon Muloi, Eric M Fèvre, Judy Bettridge, Robert Rono, Daniel Ong'are, James M Hassell, Maurice K Karani, Patrick Muinde, Bram van Bunnik, Alice Street, Margo Chase-Topping, Amy B Pedersen, Melissa J Ward, Mark Woolhouse

**Affiliations:** 1 Usher Institute of Population Health Sciences & Informatics, University of Edinburgh, Edinburgh, UK; 2 Centre for Immunity, Infection and Evolution, University of Edinburgh, Edinburgh, UK; 3 Institute of Infection and Global Health, University of Liverpool, Liverpool, UK; 4 International Livestock Research Institute, Nairobi, Kenya; 5 Nuffield Department of Clinical Medicine, University of Oxford, Oxford, UK; 6 Social Anthropology, School of Social and Political Science, University of Edinburgh, Edinburgh, UK; 7 Institute of Evolutionary Biology, School of Biological Sciences, University of Edinburgh, Edinburgh, UK; 8 The Roslin Institute, University of Edinburgh, Edinburgh, UK; * Contributed equally to this work

## Abstract

**Background:**

Antimicrobial resistance (AMR) driven by antibiotic consumption is a growing global health threat. However, data on antimicrobial consumption patterns in low- and middle-income countries (LMICs) is sparse. Here, we investigate the patterns of antibiotic sales in humans and livestock in urban Nairobi, Kenya, and evaluate the level of awareness and common behaviours related to antibiotic use and AMR amongst human and veterinary pharmacists.

**Methods:**

A total of 40 human and 19 veterinary drug store pharmacists were interviewed in Nairobi in 2018 using a standard questionnaire. Data recorded included demographic variables, types of antibiotics sold, antibiotic customers, antibiotic prescribing practices and knowledge of antibiotic use and AMR.

**Results:**

Our study shows that at the retail level, there is a considerable overlap between antibiotic classes (10/15) sold for use in both human and veterinary medicine. Whilst in our study, clinical training significantly influenced knowledge on issues related to antibiotic use and AMR and respondents had a relatively adequate level of knowledge about AMR, several inappropriate prescribing practices were identified. For example, we found that most veterinary and human drug stores (100% and 52% respectively) sold antibiotics without a prescription and noted that customer preference was an important factor when prescribing antibiotics in half of the drug stores.

**Conclusion:**

Although more research is needed to understand the drivers of antibiotic consumption in both human and animal populations, these findings highlight the need for immediate strategies to improve prescribing practices across the pharmacists in Nairobi and by extension other low- and middle-income country settings.

Antibiotic resistance (AMR) has been described as one of the most serious public health threats of this century [1-3]. Antimicrobial use, misuse and overuse in human and animal medicine exerts an important selective pressure for AMR. Global antimicrobial use in human and food animals is increasing, mainly due to increased disease burdens and expanded intensive livestock production respectively [4].

As in most cities in low and middle income countries (LMICs), in urban Nairobi the high incidence of bacterial diseases and antimicrobial resistance in clinical medicine is a major public health challenge [5]. In both human and animal populations, antibiotics are used for both prophylaxis and treatment of infectious diseases and many of the antibiotics used to treat these infectious diseases are pharmacologically similar. It is estimated that more than half of all antibiotics (for use in both humans and animals) are purchased without a prescription and used over-the-counter [6]. There is a paucity of data in Kenya regarding antibiotic usage at both the national and the regional level, but there have been attempts to assess the consumption of antibiotics in food producing animals and human health using sales data [7]. These studies, based on antibiotic import data, estimate that, from 1997-2001, consumption of antibiotics in clinical medicine increased by 4%, with penicillins and fluoroquinolones being the most widely used antibiotics. Collecting data on antibiotic use simultaneously in both animals and humans could provide essential data to help disentangle the primary drivers for the development of antibiotic resistance. Here, we carried out a survey to investigate the patterns of antibiotic sales in humans and animals in urban Nairobi.

Pharmacists (both human and veterinary) play a pivotal role in enhancing antimicrobial stewardship initiatives, not just by highlighting the AMR problem, but also by influencing crucial prescribing decisions [8,9]. To further improve antibiotic use and antibiotic stewardship programmes it is important to have an understanding of the knowledge and attitudes towards antibiotics within different populations such as pharmacists. At present, there has been limited research in understanding pharmacists’ knowledge of antibiotic resistance. Here, we aimed to assess the level of awareness and common behaviours related to antibiotic prescribing amongst human and veterinary pharmacists.

## MATERIALS AND METHODS

### Study design and setting

**Figure 1:**
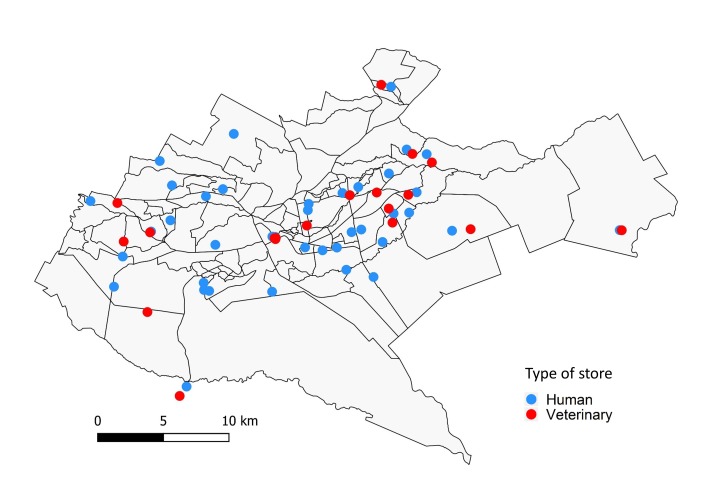
Map of Nairobi county indicating locations of study sites.

A cross-sectional study targeting human and veterinary drug stores in urban Nairobi, Kenya was carried out in January 2018 as part of the UrbanZoo project [10]. Briefly, Nairobi County was classified into seven wealth categories according to average income, identifying 70 possible sub-locations. Thirty-three sub-locations were chosen for sampling with the aim of maximising spatial distribution, socio-economic diversity, and attempting to capture the diversity of livestock keeping practices across the city [10]. Within each of the pre-selected 33 sub-locations we randomly selected and visited two community drug stores – one veterinary drug store and one human drug store. The final distribution of sampled human and veterinary drug stores is shown in **Figure 1**. Community human drug stores are mostly operated by pharmaceutical technicians who are responsible for dispensing antibiotics, while only a few, mostly large, drug stores have a registered pharmacist (holding a bachelor’s degree in pharmacy). Both pharmaceutical technicians and pharmacists are able to sell, but not prescribe antibiotics in Kenya [11]. Veterinary drugs stores are mostly operated by animal health technicians (also referred to as para-veterinarians) with just a few operated by veterinarians. Animal health technicians are also not allowed to prescribe antibiotics. All of the above-mentioned groups will have obtained clinical/veterinary training at varying levels. In this study, we define ‘pharmacist’ as someone selling antibiotics in a veterinary or a human drug store irrespective of the level of clinical training.

A draft of the questionnaire was pre-tested with five drug stores (three human and two veterinary), and refined on basis of the feedback from the pre-testing sessions before final dissemination. In each drug store a detailed questionnaire was used to collect data on socio-demographic variables, training on antibiotic use, types of antibiotics sold (by class), the four antibiotic classes most commonly sold, variation in antibiotic sales, antibiotic sources, antibiotic customer characteristics, and antibiotic prescribing practices. A summary of the collected data are presented in Table S1 in **Online Supplementary Document**. To assess the knowledge of antibiotic use and AMR amongst the human and veterinary pharmacists we adapted a standard questionnaire prepared and used by the World Health Organization [12]. First, we assessed respondents’ knowledge on a number of terms routinely used to describe the problem of antimicrobial resistance. These terms included: antibiotic resistance, superbugs, antimicrobial resistance, AMR, drug resistance and antibiotic-resistant bacteria. Next, respondents were asked about their level of agreement with ten statements describing their knowledge on AMR and potential solutions to antimicrobial resistance. The statements were written on a 5-point Likert scale [13].

### Data analysis

Descriptive statistics were prepared for all data including frequencies and percentages for categorical variables (eg, gender and education level) and means, medians, standard deviations (SDs), quartiles, and ranges for quantitative variables (eg, number of customers) depending on the distribution of the data. We used a χ^2^ or Fisher exact test using R package stats [14] to describe differences between proportions of clinical training (present or absent) by type of drug store (human or veterinary) and Mann-Whitney U test to compare range of antibiotics (number of different antibiotic classes) in the two types of drug stores.

### Prescribing practices

Next, we aimed to describe practices and evaluate the factors associated with drug prescribing amongst human and veterinary pharmacists. To achieve this, we collected data on information provided to customers after purchasing antibiotics as an indicator of good prescribing practices. This included: whether pharmacists provided customers with information on dosage, directions for use (ie, completing the prescribed dose), storage instructions, side effects, expiry date and contra-indications [15]. The data were then assessed for multicollinearity using the corrplot package [16] in R to determine if answers to any two or more questions were correlated. There did not appear to be a sufficiently strong correlation between any two questions for any of them to be excluded. To derive a measure of prescribing practices amongst the respondents we developed a composite score (sum of the binary variables, 0/1) from individual indicators of good prescribing practices. We fitted a generalized linear model (GLM) using R package lme4 [17] to assess possible influence of type of drug store (human/veterinary), clinical/veterinary training (present or absent), education level (high or low) and range of antibiotics sold (number of different antibiotic classes) in the drug store (proxy for store size) on the composite prescribing practices score. We analysed clinical/vet training (defined as having a degree or diploma in clinical or veterinary medicine) and education level separately as some pharmacists had received training in disciplines not related to medicine or veterinary studies. We considered *P* < 0.05 to be statistically significant.

### Knowledge on antimicrobial resistance

In order to assess the internal consistency of the ten statements evaluating the level of knowledge on AMR, Cronbach’s alpha coefficients were calculated for each statement. Internal consistency is a measure of item-total correlations and reliability of the scale, thus describing the extent to which all items in a test measure the same concept or construct [18]. An unstandardized Cronbach’s alpha coefficient of 0.7 or above was considered to demonstrate adequate reliability.

Principal Component Analysis (PCA) using polychoric correlation [19] was used to generate a composite index for knowledge score and to investigate clustering of the knowledge statements [20]. Analyses were performed using the psych package [21] to conduct PCA (using the principal function) without rotation of axes. Scree plot inspection [22] and parallel analysis [23] were used to choose the optimal model in terms of number of components to retain.

The scores of the first PCA component were used as measure of knowledge of AMR, and the higher the knowledge score, the higher the implied knowledge of AMR of that respondent. A generalised linear model was used to investigate the possible influence of type of drug store (human or veterinary), clinical training (present or absent), education level (high or low), and range of antibiotics sold in the drug store (proxy for store size) on the level of knowledge of AMR.

### Ethical approval

Ethical approval for this study was obtained from the International Livestock Research Institute (ILRI) Institutional Research Ethics Committee (ILRI IREC) (project reference: ILRI-IREC2017-35).

## RESULTS

### Demographic data about the respondents

A total of 59 participants were interviewed – 40 from human drug stores and 19 from veterinary drug stores (**Table 1**). Some sub-locations did not have a veterinary drug store as these tend to be located in zones of the city where animals are kept. The median age of participants in both human and veterinary stores was 30 years (range; human, 21-51; livestock, 19-67 years). More than two thirds of participants interviewed in both stores were employees (human, 73% and veterinary, 74%), and the remainder were store owners. Significantly more human pharmacists (90%) than veterinary pharmacists (57%) had undergone some form of clinical training (*P* = 0.01, Fisher exact test). Additionally, for participants who underwent clinical training, professional development programmes/trainings aimed at continuing education in AMR were an important source of information on antibiotic stewardship (human pharmacists, 50%; and veterinary pharmacists, 41%).

**Table 1:** Participant demographics and baseline clinical characteristics

Characteristic	Human drug stores	Veterinary drug stores
**Number of individuals**	40 (67%)	19 (33%)
**Gender:**		
Female	21 (52.5%)	9 (47.4%)
Male	19 (47.5%)	10 (52.6%)
**Highest education level:**		
Primary	0	1 (5.2%)
Secondary	4 (10%)	5 (26.3%)
Certificate	4 (10%)	5 (26.3%)
Diploma	24 (60%)	7 (36.8%)
Degree	8 (20%)	1 (5.3%)
**Role:**		
Owner	11 (27.5%)	5 (26.3%)
Worker	29 (72.5%)	14 (73.7)
**Age (median)**	30	30
**Clinical/veterinary training:**		
Present	36 (90%)	11 (57%)
None	4 (10%)	8 (42.1%)
**Source of training on antibiotic stewardship and AMR:**		
Clinical training only	16 (40%)	3 (15.8%)
CPD	20 (50%)	8 (41.1%)
None	4 (10%)	8 (42.1%)

AMR – antimicrobial resistance, CPD – continuing professional development

### Antibiotics sold and sale dynamics

**Figure 2:**
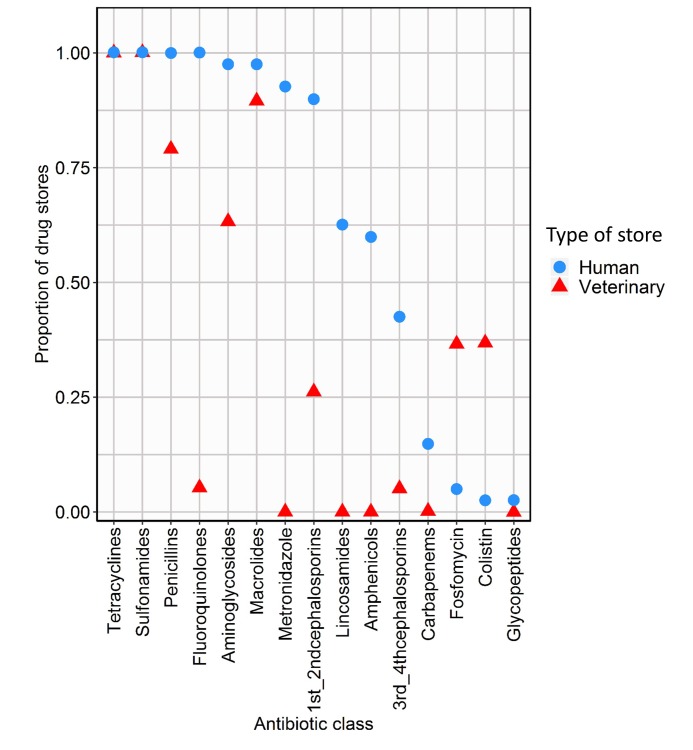
Proportion of the 15 antibiotic classes reported in human (n = 40) and/or veterinary drug stores (n = 19). Data arranged in order of the average proportion of antibiotic classes.

A total of 15 antibiotic classes were available in either or both human and veterinary drug stores (**Figure 2**). Two thirds of the antibiotic classes (10/15) were found in both human and veterinary drug stores while five classes (metronidazole, amphenicols, lincosamides, glycopeptides and carbapenems) were only found in human drug stores. Of the ten overlapping antibiotic classes, beta lactam/penicillin, tetracycline, sulfonamide, and macrolide antibiotic classes were found in more than 78% of both types of drug stores. Of note, carbapenems, third and fourth generation-cephalosporins and glycopeptides – antibiotics restricted to clinical use – were found in 15%, 4% and 3% of human drug stores respectively. Overall, human drug stores had a broader range of antibiotics available for sale when compared to veterinary stores (*P* < 0.01, Mann-Whitney U test) (**Figure 2**).

**Figure 3:**
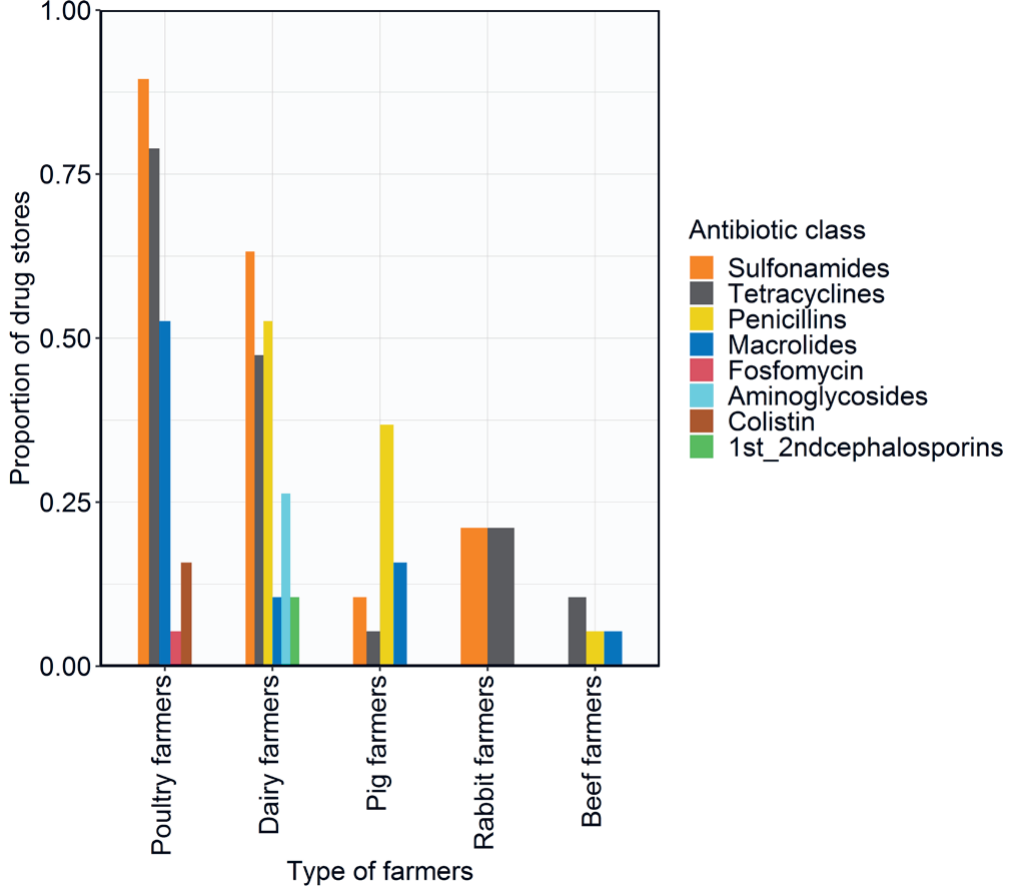
Proportion of drug stores reporting the most commonly purchased antibiotics by different types of farmers based on the primary animal on the farm.

Penicillins, metronidazole, fluoroquinolones, and first and second-generation cephalosporins were reported as being amongst the four most commonly sold antibiotic classes by the human drug stores in 93%, 65%, 63%, and 43% of the stores respectively. However, among the veterinary drug stores, tetracyclines, sulfonamides, penicillins and macrolides were reported to be amongst the four most commonly sold antibiotic classes in 79%, 74%, 58%, and 47% of the stores respectively. Tetracyclines and sulfonamides were reported to be amongst the four most commonly purchased antibiotic classes by poultry farmers in 79% and 90% of the veterinary drug stores respectively. The antibiotic colistin was described as being commonly purchased by poultry farmers in 16% of the drugs stores. Sulfonamides, tetracyclines and penicillins were reported to be amongst the four most commonly purchased antibiotic classes by dairy farmers in 63%, 47% and 52% of drug stores respectively. In 11% of the veterinary drug stores, dairy farmers reportedly purchased first and second-generation cephalosporin intra-mammary tubes to treat mastitis cases. The antibiotics reported to be amongst the four most commonly purchased antibiotic classes by pig farmers were penicillins, macrolides and sulphonamides in 37%, 16% and 11% of the veterinary drug stores respectively (**Figure 3**).

Fifty eight percent of human and 42% of veterinary drug stores reported a rise in antibiotic sales compared to the same period a year earlier. Increased customer demand for antibiotics was believed to be the main driver by 80% and 60% of human and veterinary pharmacists respectively. Wholesale operations (defined as companies that buy drugs in bulk and sell them in smaller quantities to drug stores) were reported as the main provider of antibiotics to human drug stores (78%). On the other hand, distribution companies (defined as corporations that purchase drugs from pharmaceutical companies, store and subsequently distribute to drug stores) were reported as the main provider of antibiotics to veterinary drug stores (58%).

### Antibiotic customer characteristics

The average daily number of customers purchasing antibiotics was not significantly different (*P* = 0.20; Mann-Whitney U test) between human drug stores (25 customers, range 2-130) and veterinary drug stores (14 customers, range 2-113).

Antibiotics were reportedly prescribed frequently for respiratory tract infections, gastro-intestinal infections, and sore throat in 83%, 65% and 58% of human drug stores respectively. Additional prescriptions were linked to fever, body aches, and skin wounds in 38%, 35% and 13% of human drug stores respectively.

Poultry farmers and veterinary para-professionals were the most frequent customers of antibiotics, being reported as customers in 95% and 74% of the veterinary drug stores respectively. Other customers for antibiotics included: dairy farmers, veterinarians, pig farmers, rabbit famers, and beef farmers in 63%, 58%, 47%, 37%, and 11% of stores.

### Knowledge of antimicrobial resistance

**Figure 4:**
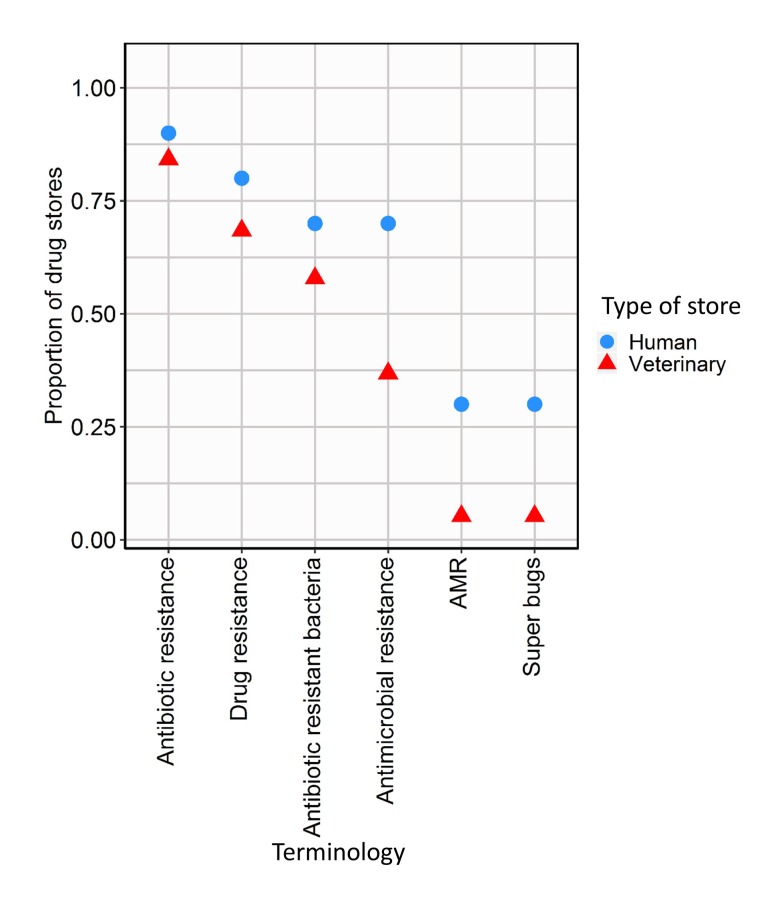
Terms used to describe antimicrobial resistance.

More than two-thirds of the respondents in both stores types were aware of the terms “antibiotic resistance” and “drug resistance”. By contrast, fewer than half of respondents had heard of the terms “AMR” and “super bugs” (**Figure 4**).

More than three quarters of the respondents in both store types agreed that the prevalence of drug resistant infections was increasing, and if left unchecked routine medical and surgical procedures would become a much riskier proposition. Likewise, more than 79% in both types of stores recognised that AMR is a problem, and has the potential to affect any country and anyone, including them and/or their families. However, most respondents (>80%) believed that AMR occurs when their body becomes resistant to antibiotics rather than the bacteria themselves that develop resistance. Similarly, 40% and 53% of human and veterinary respondents respectively suggested that AMR is only a problem for regular consumers of antibiotics. More than half (52%) of the respondents interviewed responded neutrally or disagreed with the statement that antibiotic resistant bacteria could be spread from person to person (Table S3 and Figure S2 in **Online Supplementary Document**).

### Association between sociodemographic factors and knowledge on AMR

Cronbach’s alpha coefficient for the ten “knowledge statements” was 0.74, suggesting an acceptable level of internal consistency and a potential underlying latent construct (Table S2 and Figure S1 in **Online Supplementary Document**). The knowledge score (knowledge about AMR) of the respondents had significant positive association with medical/veterinary training (*P* = 0.02), meaning respondents with clinical training had a higher knowledge about AMR than those who had not undergone such training. The level of knowledge about AMR did not differ by store type, education level or range of antibiotics available in the store (*P* > 0.05) (**Table 2**).

**Table 2:** Results of a multivariable regression examining the influence of sociodemographic factors on knowledge about AMR in a sample of 40 and 19 human and veterinary drug stores respectively.

Variable	Estimate	Standard error	χ^2^	df	*P*-value
Veterinary drug store	0.07	0.02	0.3	1	0.59
Range of antibiotics	-0.01	0.02	0.31	1	0.58
Clinical/veterinary training	0.3	0.14	4.86	1	0.02
High education level	0.04	0.12	0.13	1	0.72

### Knowledge and views on potential solutions to AMR

More than 80% of respondents in both store types agreed that people should use antibiotics only when prescribed by a medical practitioner. Also, more than two thirds of respondents in both store types agreed that reducing antibiotic use in food animals could help address the problem of antibiotic resistance. In both store types, respondents agreed on the need for governments and pharmaceutical companies to invest in research and development of new antibiotics. More than 84% of all respondents agreed that everyone should use antibiotics prudently, but more than 73% of respondents thought that medical experts would solve the problem of antibiotic resistance. Hand washing and vaccination of children against infections were supported by more than 94% of respondents in both store types. However, 38% and 26% of respondents in human and veterinary drug stores agreed that there was not much they could do to stop antibiotic resistance (Table S4 and Figure S3 in **Online Supplementary Document**).

### Antibiotic prescribing practices

Fifty-two per-cent (21/40) of the human drug stores reported that they sold antibiotics without a prescription while all veterinary drug stores sold antibiotics without a prescription. Multivariable logistic regression analysis revealed prescribing practices did not vary significantly by clinical training, store type, range of antibiotics sold and/or education level (*P* > 0.05) (Table S5 in the **Online Supplementary Document**).

Across both human and veterinary drug stores, the most important factor for prescribing antibiotics was indication of use – based on symptoms – (in >75% of the stores), followed by price of the antibiotic (in >50% of the stores). Of note, 28% and 31% of human and veterinary pharmacists respectively considered customer preference as an important factor when prescribing an antibiotic.

## DISCUSSION

In this study, we aimed to investigate the patterns of antibiotic sales in humans and animals in a large and rapidly developing city in a LMIC: Nairobi, Kenya. We also evaluated the level of awareness and common behaviours related to antibiotic use and AMR amongst human and veterinary pharmacists. Our study was based on gathering antibiotic sales data from human and veterinary drug stores across the city, where sales data were interpreted as representing antibiotic usage.

Our study shows considerable overlap in the antibiotic classes (10/15) sold for human and animal use in urban Nairobi, with marked variations in the sale of some antibiotic classes such as cephalosporins and fluoroquinolones – mostly found in human drug stores. This overlap in antibiotic classes, including of critically important antimicrobials [24], highlights the need for prudent use of all antimicrobials and continued monitoring and surveillance of antimicrobial usage in LMIC urban settings [25].

The most common symptoms prompting antibiotic purchase in humans were similar to those reported in other studies, respiratory tract infections and diarrhoeal disease [7,26,27]. Broad-spectrum beta lactams, fluoroquinolones, first and second-generation cephalosporins and metronidazole were the most commonly sold/bought antibiotics in human drug stores. This finding is consistent with antibiotic prescription in the community in previous Kenyan studies [27,28], in other low income countries such as Uganda [29], Tanzania [30], India [31] and in high income countries such as United Kingdom [32] and the USA [33]. Our finding that WHO-classified highest priority critically important antibiotic classes such as carbapenems, third and fourth generation cephalosporins, and glycopeptides were sold over the counter and potentially without prescription in human drugs stores is of public health concern.

In the current study, tetracyclines, sulphonamides, penicillins, and macrolides were the most commonly purchased veterinary antibiotics and poultry farmers were the major consumers of antibiotics. Further, our findings indicate that colistin – a drug considered of last resort in human medicine [34] – was an antibiotic of choice amongst poultry farmers in 16% of veterinary drug stores, as has been found in previous studies in other parts of the world [35-37]. Urban livestock are increasingly important, particularly among the low and middle income population bracket in most low resource urban settings [38,39], and antibiotic usage is a low-cost alternative for comprehensive hygiene and biosafety measures [40].

Knowledge about antimicrobial resistance among pharmacists has only been studied to a limited extent in LMICs [41]. Consistent with a recent multi-country survey by the World Health Organization [12] our survey found that, whilst the majority of the pharmacists have an understanding of the problem of antibiotic resistance and the effect(s) on public health, they do not fully understand how AMR develops and spreads. Encouragingly, the majority of respondents (>80%) identified several behaviours that can help reduce AMR burden; such as handwashing, antibiotic stewardship by both doctors and the public, and ensuring children’s vaccinations are up-to-date. However, considering their key role in antibiotic stewardship, the finding that 38% and 26% of human and veterinary pharmacists agreed there was little they could do to stop AMR highlights the need for enhanced involvement of pharmacists in antibiotic stewardship programs.

Whilst the majority of the pharmacists we interviewed have an understanding of the threat posed by AMR to public health, our data highlight the poor quality of community pharmacy practice, most notably the dispensing of antibiotics without prescriptions and the inclusion of customer preference as an important factor when selling antibiotics. Antibiotics were dispensed without prescription in 53% and 100% of the human and veterinary drug stores respectively; a finding consistent with similar studies in Tanzania (92.3%) [42], Serbia (47.2%) [43], Ghana (70%) [44], and broadly across the developing world (19%-100%) [25]. By contrast, a recent study conducted in community pharmacies in Nairobi reported low sale of antibiotics without prescription [45]. Part of this difference, however, may be related to the fact that in that study, information was based on just three pharmacies hence not generalizable across the city. In our study, whilst clinical training significantly influenced knowledge on issues related to antibiotic use and AMR, prescribing practices did not change with levels of clinical training. Considering the complexity of factors contributing to antibiotic prescribing, including the public’s demand for antibiotics, behavioural and policy interventions could be explored [46]. Because many members of the public in most LMICs bypass health care facilities and veterinarians in favour of seeking medication at pharmacies, policy makers could consider expanding the role of pharmacists in antibiotic stewardship initiatives [47,48].

Restating the particular relevance of training to antibiotic stewardship measures, the role of enhanced training in antimicrobial prescribing and AMR has been identified in surveys of both medical personnel and the public, both in Kenya and globally [49]. Results from a recent survey indicate that only 14.1% of clinicians in a national referral and teaching hospital in Kenya had received more than four lectures on antimicrobial stewardship and AMR as part of their medical training [50]. To address this challenge, antibiotic stewardship needs to be integrated in the undergraduate veterinary/medical curriculums and continuing medical/veterinary education programs.

Similar to other studies [51,52], our findings indicate greater familiarity amongst human and veterinary pharmacists with ‘antibiotic resistance’ and ‘drug resistance’ terminologies, and minimal familiarity with ‘AMR’ and ‘superbugs’. This indicates that public health initiatives on antibiotic stewardship and/or antimicrobial resistance initiatives need to take an evidence-based approach in designing effective communication strategies [51,53].

This is the first study designed to capture the overlapping patterns of antibiotic sales in humans and livestock in a developing city via an epidemiologically-structured approach. A variety of approaches are available for assessing patterns of antibiotic use in humans and animals [54]. Considering that pharmacies are the primary level of outpatient/veterinary care (consultation, diagnosis, and prescription of antibiotics) for many urban dwellers in Nairobi, focusing on them provides important insights into the probable antibiotic usage patterns at the consumer level. Future research would benefit from conducting longitudinal surveys of antimicrobial use in health care facilities and the community to better assess trends over time. While, we acknowledge that our study used a relatively small sample size (19 and 40 veterinary and human drug stores respectively), we did not find much heterogeneity in the results obtained. It is important to highlight that although extrapolating antimicrobial consumption from sales data are not ideal, and care will be required when interpreting our results, various other studies have shown that relying on sales data are of direct relevance for initiatives aimed at monitoring global and national antimicrobial patterns [55,56]. Although this study focused on pharmacists in urban Nairobi, these results are likely to be relevant to many other developing cities across the world with large income disparity and where livestock are commonly kept in close contact with humans.

## CONCLUSIONS

Monitoring and surveillance of antibiotic use in LMICs is challenging, but vital, as it provides valuable information for public health policy. Our study shows that at the retail level in urban Nairobi, there is a considerable overlap between antibiotic classes available for use in both human and veterinary medicine. Whilst the majority of human and veterinary pharmacists showed high knowledge about antibiotic use and antimicrobial resistance, inappropriate prescribing practices were noted, highlighting the need for continued education to the pharmacists and the public, about prudent antibiotic prescribing and use. Although further research is necessary to understand the drivers of antibiotic consumption in both populations, it is clear that interventions are urgently required to improve prescribing practices across the pharmacists.

## Additional material

Online Supplementary Document
