# Supplementary material for: A cross-sectional survey of practices and knowledge among antibiotic retailers in Nairobi, Kenya
Source: J Glob Health. 2019 Aug 25;9(2):020412. doi: 10.7189/jogh.09.020412 (PMC6708591; doi:10.7189/jogh.09.020412)

Supplementary material - A cross-sectional survey of practices and knowledge among antibiotic retailers in Nairobi, Kenya

Table s1: Summary of variables

Theme	Data collected	Data description
Socio-demographic variables	Type of drug store	• Human<br>• Veterinary
	Age	Years
	Gender	• Male<br>• Female
	Educational level	High<br>• Doctorate degree<br>• Master's degree<br>• Bachelor's degree<br>• College (Certificate/Diploma/Higher Diploma)<br>Low<br>• No formal Education<br>• Primary Education<br>• Secondary Education
	Number of workers in the drug store	Number
	Type of business	• Sole proprietorship<br>• Joint ownership<br>• Chain store
	Respondents role in the drug store	• Owner<br>• Full time employee<br>• Part time employee
	Duration of working in the drug store	• More than 5 years<br>• 2-4 years<br>• Less than 2 years
	Training in medical/veterinary sciences	• Bachelor's degree (Medicine, pharmacy, vet medicine nursing etc.)<br>• Diploma (clinical medicine, pharmacy, animal health etc.)<br>• Certificate (clinical medicine, pharmacy, nursing etc.)<br>• None
	Received specific training on appropriate use of antibiotics	Categorical (yes/no)
	Source of training	• Degree/diploma training more than 3 years ago<br>• Degree/diploma training less than 3 years ago<br>• Continuous development program/workshop (> 3 years ago)<br>• Continuous development program/workshop (< 3 years ago)
Antibiotics sold and sale dynamics	Antibiotics available in the store for sale	20 antibiotic classes as defined by World Health Organization List of Essential Medicines
	Common sold antibiotic classes (top 4)	Participants were asked to subjectively describe which 4 antibiotic classes were most commonly sold
	Antibiotic sale changes compared to similar period in the previous year	• Increased<br>• Decreased<br>• No change
	Reasons for change in antibiotic sales	• More/less demand from customers<br>• More/less institutional procurement procedures<br>• More/less supply coming from suppliers
Antibiotic supply	Sources of antibiotics	• Neighbouring wholesaler<br>• Wholesaler in a another location in Nairobi county<br>• Wholesaler in a another location outside Nairobi county<br>• Drug distribution company<br>• Drug manufacturing company<br>• Import from an international source
	Important factors when purchasing antibiotics	• Price<br>• Service (i.e. reliability)<br>• Product quality (trusted brand)
	Number of customers purchasing antibiotics	Number

Antibiotic prescribing practices and customer characteristics	Number of customers purchasing antibiotics without a prescription	Number
	Antibiotic classes sold without needing a written prescription	Number
	Common presented complaints for purchasing antibiotics (human drug stores)	
	Commonly sold antibiotics to different types of farmers/customers (veterinary drugs stores)	
	Information provided to customers when buying antibiotics	• Correct dosage<br>• Directions for use<br>• Storage instructions<br>• Potential side effects<br>• Expiry date<br>• Contra-indications
	Factors taken into account when recommending a certain antibiotic to a customer	• Price of antibiotic<br>• Type of antibiotic<br>• Availability of antibiotic<br>• Indications of use<br>• Efficacy<br>• Adverse reactions/effects<br>• Recommended antibiotic choice<br>• Customer preference
Knowledge on antimicrobial resistance	Statements about knowledge on antimicrobial resistance	• Antibiotic resistance occurs when your body becomes resistant to antibiotics and they no longer work as well<br>• Many infections are becoming increasingly resistant to treatment by antibiotics<br>• If bacteria are resistant to antibiotics, it can be very difficult or impossible to treat the infections they cause<br>• Antibiotic resistance is an issue that could affect me or my family<br>• Antibiotic resistance is only a problem for people who take antibiotics regularly<br>• Bacteria which are resistant to antibiotics can be spread from person to person<br>• Antibiotic-resistant infections could make medical procedures like surgery, organ transplants and cancer treatment much more dangerous<br>• Antibiotic resistance is one of the biggest problems the world faces<br>• Antibiotic resistance is one of the biggest problems Kenya faces<br>• I am not at risk of getting an antibiotic resistant infection, as long as I take my antibiotics correctly
	Statements about potential solutions to AMR	• There is not much people like me can do to stop antibiotic resistance<br>• People should use antibiotics only when they are prescribed by a doctor or nurse<br>• Farmers should give fewer antibiotics to food-producing animals<br>• People should not keep antibiotics and use them later for other illnesses<br>• The governments should reward the development of new antibiotics<br>• Pharmaceutical companies should develop new antibiotics<br>• Doctors should only prescribe antibiotics when they are needed<br>• Medical experts will solve the problem of antibiotic resistance before it becomes too serious<br>• Everyone needs to take responsibility for using antibiotics responsibly<br>• Parents should make sure all of their children’s vaccinations are up-to-date<br>• People should wash their hands regularly

Table s2. Cronbach's alpha for responses to ten statements about knowledge on antimicrobial resistance

Knowledge statement	Raw alpha	Std. alpha	G6	Average R	S/N	alpha se
Antibiotic resistance occurs when your body becomes resistant to antibiotics and they no longer work as well	0.74	0.75	0.81	0.25	3	0.05
Many infections are becoming increasingly resistant to treatment by antibiotics	0.72	0.74	0.79	0.24	2.8	0.056
If bacteria are resistant to antibiotics, it can be very difficult or impossible to treat the infections they cause	0.7	0.71	0.78	0.22	2.5	0.059
Antibiotic resistance is an issue that could affect me or my family	0.69	0.7	0.74	0.2	2.3	0.061
Antibiotic resistance is only a problem for people who take antibiotics regularly	0.69	0.72	0.78	0.22	2.5	0.06
Bacteria which are resistant to antibiotics can be spread from person to person	0.69	0.71	0.78	0.22	2.5	0.061
Antibiotic-resistant infections could make medical procedures like surgery, organ transplants and cancer treatment much more dangerous	0.69	0.71	0.78	0.22	2.5	0.06
Antibiotic resistance is one of the biggest problems the world faces	0.69	0.7	0.76	0.2	2.3	0.06
Antibiotic resistance is one of the biggest problems Kenya faces	0.67	0.68	0.75	0.19	2.1	0.063
I am not at risk of getting an antibiotic resistant infection, as long as I take my antibiotics correctly	0.73	0.75	0.8	0.25	3	0.053

* G6: Guttman's lambda 6 - calculated from the squared multiple correlation. Average R: average inter-item correlation.

Figure s1. Principal component analysis of attitudes and perceptions related to antimicrobial resistance, from a sample of 40 human pharmacists and 19 veterinary based on the responses to ten statements about knowledge on antimicrobial resistance.

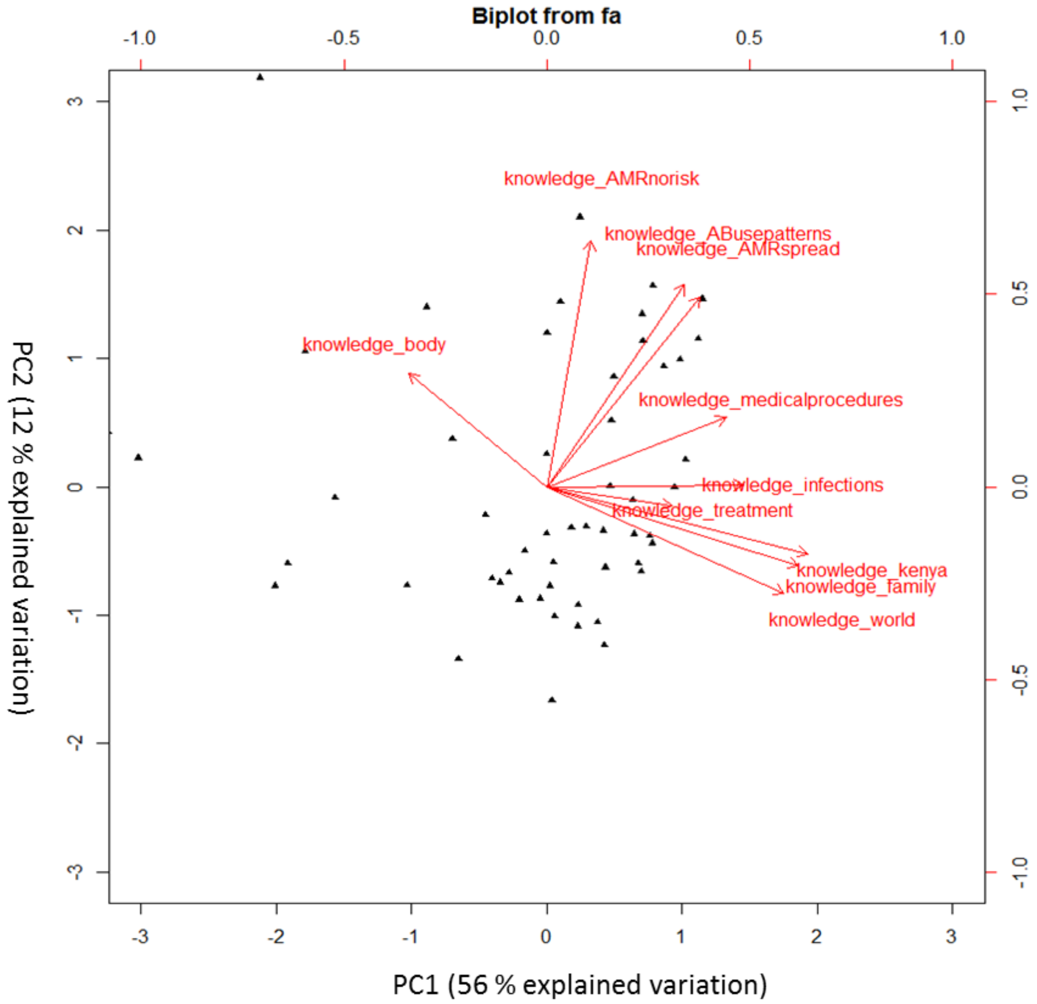

knowledge_body	Antibiotic resistance occurs when your body becomes resistant to antibiotics and they no longer work as well
knowledge_treatment	Many infections are becoming increasingly resistant to treatment by antibiotics
knowledge_infections	If bacteria are resistant to antibiotics, it can be very difficult or impossible to treat the infections they cause
knowledge_family	Antibiotic resistance is an issue that could affect me or my family
knowledge_ABusepatterns	Antibiotic resistance is only a problem for people who take antibiotics regularly
knowledge_AMRspread	Bacteria which are resistant to antibiotics can be spread from person to person
knowledge_medicalprocedures	Antibiotic-resistant infections could make medical procedures like surgery, organ transplants and cancer treatment much more dangerous
knowledge_world	Antibiotic resistance is one of the biggest problems the world faces
knowledge_kenya	Antibiotic resistance is one of the biggest problems Kenya faces
knowledge_AMRnorisk	I am not at risk of getting an antibiotic resistant infection, as long as I take my antibiotics correctly

Table s3. Percentage of responses (strongly agree/agree, strongly disagree/disagree and neutral) from 40 human and 19 veterinary pharmacists to nine statements about knowledge on antimicrobial resistance.

Knowledge and awareness of AMR	Strongly agree/ agree		Neutral		Strongly Disagree/ Disagree	
	Human (n=40)	Veterinary (n=19)	Human (n=40)	Veterinary (n=19)	Human (n=40)	Veterinary (n=19)
1. Antibiotic resistance occurs when your body becomes resistant to antibiotics and they no longer work as well	80% (32)	89.5% (17)	5% (2)	5.3% (1)	15% (6)	10.6% (2)
2. Many infections are becoming increasingly resistant to treatment by antibiotics	87.5% (35)	89.5% (17)	5% (2)	5.3% (1)	7.5% (3)	5.3% (1)
3. If bacteria are resistant to antibiotics, it can be very difficult or impossible to treat the infections they cause	80% (32)	79% (15)	5% (2)	5.3% (1)	15% (6)	15.8% (3)
4. Bacteria which are resistant to antibiotics can be spread from person to person	52.5% (21)	52.7% (10)	5% (2)	26.4% (5)	42.5% (17)	21.1% (4)
5. Antibiotic-resistant infections could make medical procedures like surgery, organ transplants and cancer treatment much more dangerous	77.5% (31)	73.7% (14)	15% (6)	15.8% (3)	7.5% (3)	10.6% (2)
6. Antibiotic resistance is one of the biggest problems the world faces	87.5% (35)	79% (15)	7.5% (3)	5.3% (1)	5% (2)	15.8% (3)
7. Antibiotic resistance is one of the biggest problems Kenya faces	85% (34)	79% (15)	7.5% (3)	5.3% (1)	7.5% (3)	15.8% (3)
8. Antibiotic resistance is an issue that could affect me or my family	92.5% (37)	89.5% (17)	0	0	7.5% (3)	5.3% (1)
9. Antibiotic resistance is only a problem for people who take antibiotics regularly	40% (16)	52.7% (10)	5% (2)	5.3% (1)	55% (22)	42.2% (8)
10. I am not at risk of getting an antibiotic resistant infection, as long as I take my antibiotics correctly	72.5% (29)	84.2% (16)	7.5% (3)	5.3% (1)	20% (8)	10.5% (2)

Figure s2. Percentage of responses on ten statements about knowledge on antimicrobial resistance from a) 40 human pharmacists b) 19 veterinary interviewed

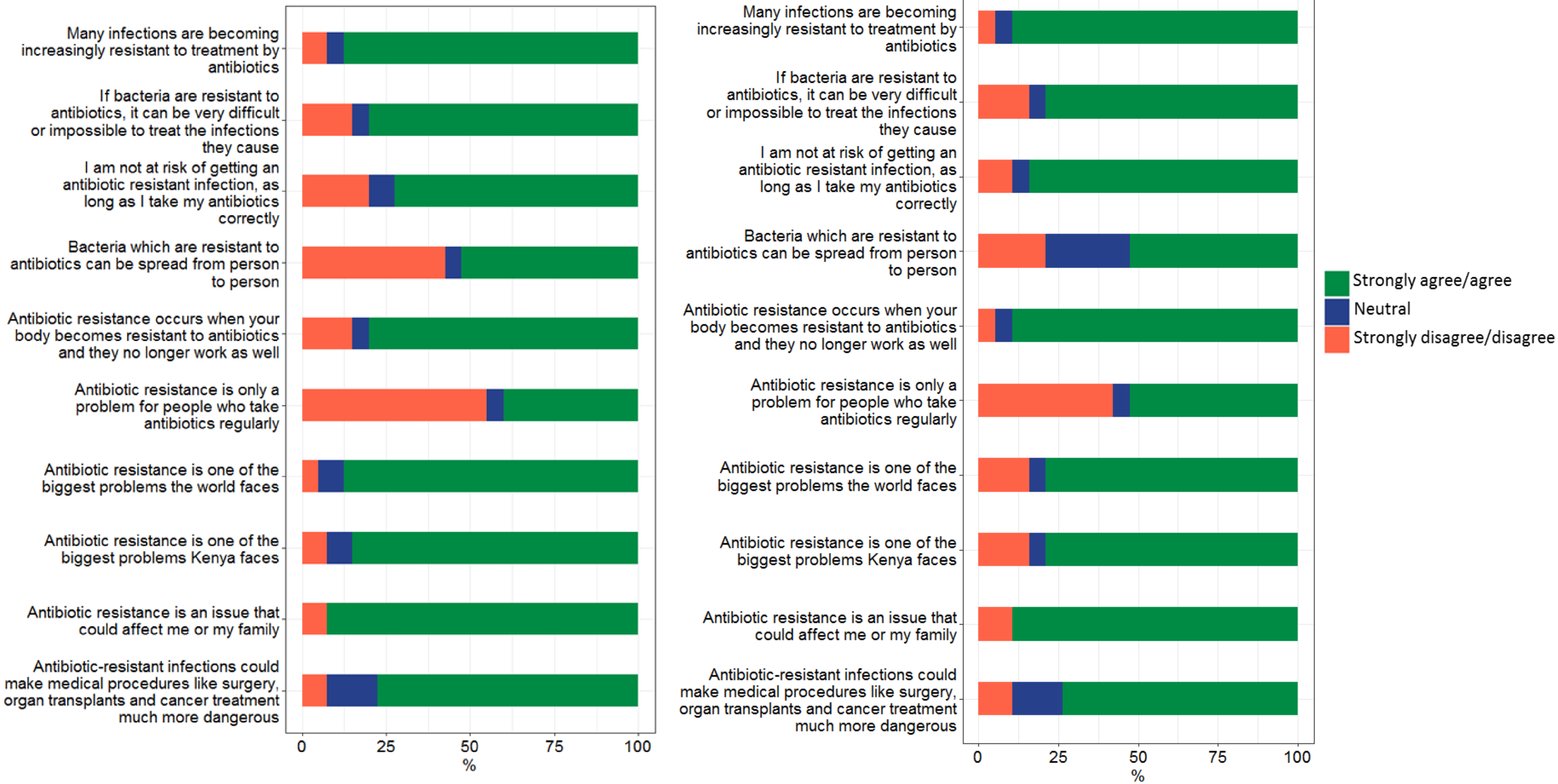

Table s4. Percentage of responses (agree, disagree and neutral) from 40 human and 19 veterinary pharmacists to ten statements on potential solution to AMR.

	Strongly agree/ agree		Neutral		Strongly Disagree/ Disagree	
**Perceptions about potential solutions to AMR**	Human (n=40)	Veterinary (n=19)	Human (n=40)	Veterinary (n=19)	Human (n=40)	Veterinary (n=19)
1. There is not much people like me can do to stop antibiotic resistance	37.5% (15)	26.4% (5)	10% (4)	10.6% (2)	52.5% (21)	63.2% (12)
2. People should use antibiotics only when they are prescribed by a doctor or nurse	82.5% (33)	94.8% (18)	2.5% (1)	5.3% (1)	15% (6)	0
3. Farmers should give fewer antibiotics to food-producing animals	67.5% (27)	79% (15)	17.5% (7)	0	15% (6)	21.1% (4)
4. People should not keep antibiotics and use them later for other illnesses	80% (32)	84.3% (16)	7.5% (3)	0	12.5% (5)	15.8% (3)
5. The governments should reward the development of new antibiotics	82.5% (33)	73.7% (14)	10% (4)	21.1% (4)	7.5% (3)	5.3% (1)
6. Pharmaceutical companies should develop new antibiotics	67.5% (27)	79% (15)	12.5% (5)	21% (4)	20% (8)	0
7. Doctors should only prescribe antibiotics when they are needed	95% (38)	94.8% (18)	5% (2)	0	0	5.3% (1)
8. Medical experts will solve the problem of antibiotic resistance before it becomes too serious	77.5% (31)	73.7% (14)	7.5% (3)	21.1% (4)	15% (6)	5.3% (1)
9. Everyone needs to take responsibility for using antibiotics responsibly	90% (36)	84.3% (16)	2.5% (1)	5.3% (1)	7.5% (3)	10.6% (2)
10. Parents should make sure all of their children's vaccinations are up-to-date	95% (38)	94.7% (18)	0	0	5% (2)	5.3% (1)
11. People should wash their hands regularly	97.5% (39)	94.7% (18)	0	0	2.5% (1)	5.3% (1)

Table s5. Results of a multivariable regression examining the influence of sociodemographic factors on prescribing practices in a sample of 40 and 19 human and veterinary drug stores respectively.

Variable	Estimate	Standard error	X^2	df	p-value
Veterinary drug store	-0.11	0.5	0.2	1	0.66
Range of antibiotics	-0.1	0.24	0.2	1	0.62
Medical/vet training	0.38	0.25	2.4	1	0.12
High education level	-0.14	0.2	0.5	1	0.49

Figure s3. Percentage of responses on 11 statements about potential solutions to AMR from a) 40 human b) 19 veterinary pharmacists

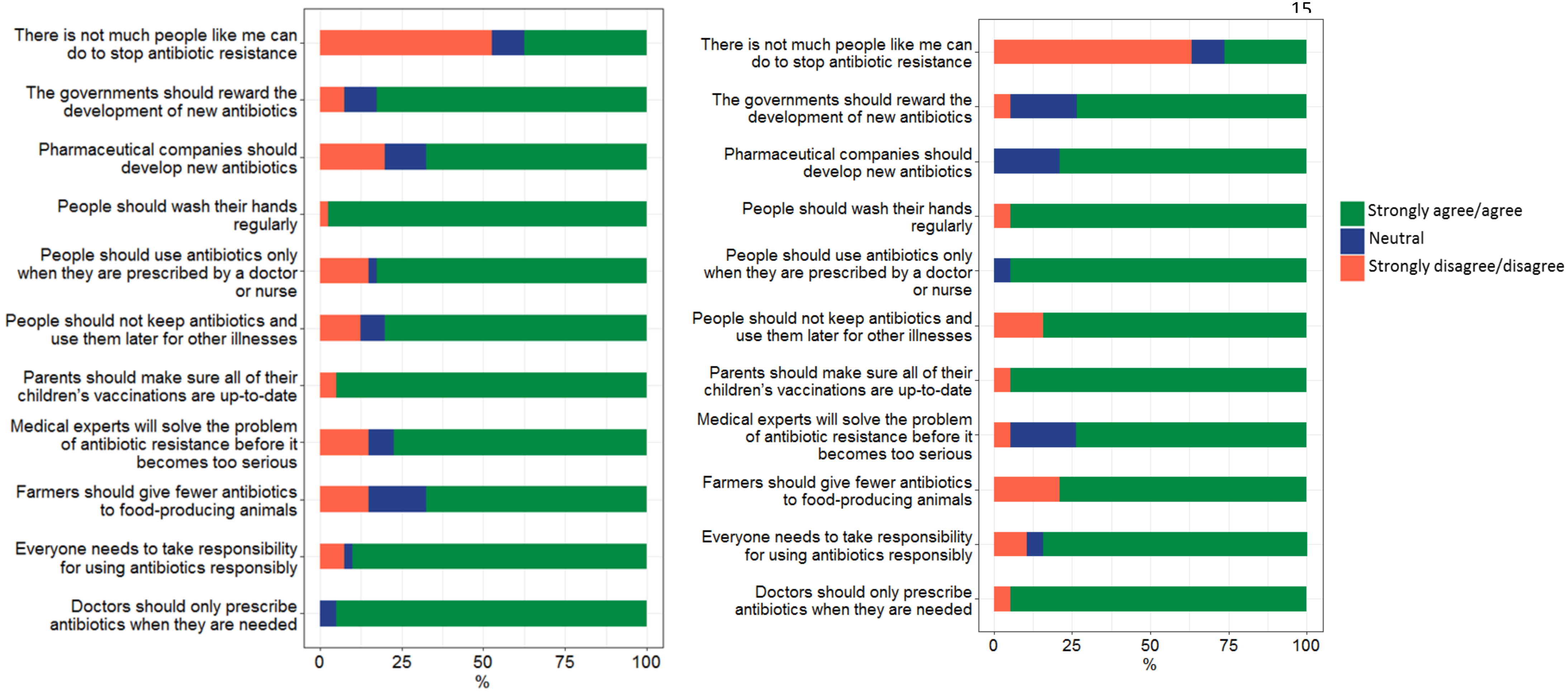

Supplement: Online Supplementary Document [file jogh-09-020412-s001.pdf]
